# Traditional and Modern Uses of Natural Honey in Human Diseases: A Review

**Published:** 2013-06

**Authors:** Tahereh Eteraf-Oskouei, Moslem Najafi

**Affiliations:** 1 Biotechnology Research Center, Tabriz University of Medical Sciences, Tabriz, Iran; 2 Department of Pharmacology and Toxicology, Faculty of Pharmacy, Tabriz University of Medical Sciences**, **Tabriz, Iran

**Keywords:** Honey, Human Diseases, Traditional Medicine, Modern Medicine

## Abstract

Honey is a by-product of flower nectar and the upper aero-digestive tract of the honey bee, which is concentrated through a dehydration process inside the bee hive. Honey has a very complex chemical composition that varies depending on the botanical source. It has been used both as food and medicine since ancient times. Human use of honey is traced to some 8000 years ago as depicted by Stone Age paintings. In addition to important role of natural honey in the traditional medicine, during the past few decades, it was subjected to laboratory and clinical investigations by several research groups and it has found a place in modern medicine. Honey has been reported to have an inhibitory effect on around 60 species of bacteria, some species of fungi and viruses. Antioxidant capacity of honey is important in many disease conditions and is due to a wide range of compounds including phenolics, peptides, organic acids, enzymes, and Maillard reaction products. Honey has also been used in some gastrointestinal, cardiovascular, inflammatory and neoplastic states. This review covers the composition, physico-chemical properties and the most important uses of natural honey in human diseases.

## Introduction

Honey is a natural product that has been widely used for its therapeutic effects. It has been reported to contain about 200 substances. Honey is composed primarily of fructose and glucose but also contains fructo-oligosaccharides (1) and many amino acids, vitamins, minerals and enzymes (2). The composition of honey varies depending on the plants on which the bee feeds. However, almost all natural honey contains flavonoides (such as apigenin, pinocembrin, kaempferol, quercetin, galangin, chrysin and hesperetin), phenolic acids (such as ellagic, caffeic, p-coumaric and ferulic acids), ascorbic acid, tocopherols, catalase (CAT), superoxide dismutase (SOD), reduced glutathione (GSH), Millard reaction products and peptides. Most of those compound works together to provide a synergistic antioxidant effect (3-7).

Honey has had a valued place in traditional medicine for centuries (8, 9). However, it has a limited use in modern medicine due to lack of scientific support (10). For a long time, it has been observed that honey can be used to overcome liver, cardiovascular and gastrointestinal problems (11). Ancient Egyptians, Assyrians, Chinese, Greeks and Romans employed honey for wounds and diseases of the intestine (12). Since a few decades ago, honey was subjected to laboratory and clinical investigations by several research groups. The most remarkable discovery was antibacterial activity of honey that has been mentioned in numerous studies (13, 14). Natural honey exhibits bactericidal activity against many organisms including *Salmonella,* *Shigella*, *Escherichia coli *(3, 15), *Helicobacter pylori* (9), etc. In an inflammatory model of colitis, honey was as effective as prednisolone treatment (16]. Research has also indicated that honey may possess anti-inflammatory activity and stimulate immune responses within a wound (17, 18). Al-Waili and Boni (2003) demonstrated anti-inflammatory effects of honey in human after ingestion of honey (19). Honey, interestingly, has been shown to prevent reactive oxygen species (ROS)-induced low density lipoprotein (LDL) oxidation in some *in vitro* studies, thus exhibiting beneficial cardiovascular protection (20, 21). Honey also had antineoplastic activity in an experimental bladder cancer (22). This article has reviewed important traditional and modern uses of natural honey in human diseases. 

*Chemical composition of natural honey*

Natural honey contains about 200 substances, including amino acids, vitamins, minerals and enzymes, but it primarily contains sugar and water. Sugar accounts for 95–99% of honey dry matter. The principal carbohydrate constituents of honey are fructose (32.56 to 38.2%) and glucose (28.54 to 31.3 %), which represents 85–95% of total sugars that are readily absorbed in the gastrointestinal tract (23, 11).

Other sugars include disaccharides such as maltose, sucrose, isomaltose turanose, nigerose, meli-biose, panose, maltotriose, melezitose. A few oligosaccharides are also present. Honey contains 4 to 5% fructooligosaccharides, which serve as probiotic agents (1, 11). Water is the second most important component of honey. Organic acids constitute 0.57% of honey and include gluconic acid which is a by product of enzymatic digestion of glucose. The organic acids are responsible for the acidity of honey and contribute largely to its characteristic taste (24). The concentration of mineral compounds ranges from 0.1% to 1.0 %. Potassium is the major metal, followed by calcium, magnesium, sodium, sulphur and phosphorus. Trace elements include iron, copper, zinc and manganese (25-27). 

Nitrogenous compounds, vitamins C, B_1_ (thiamine) and B_2_ complex vitamins like riboflavin, nicotinic acid, B_6 _and panthothenic acid are also found (24). Honey contains proteins only in minute, 0.1–0.5 percent quantities (28, 29). According to a recent report, specific protein quantities differ according to the honeybee origin (30). The average composition of honey is given in Table 1 (31).

A variety of enzymes such as oxidase, invertase, amylase, catalase, etc. are present in honey. However, the main enzymes in honey are invertase (saccharase), diastase (amylase) and glucose oxidase. They have an important role in the formation of honey (24). The enzyme glucose oxidase produces hydrogen peroxide (which provides antimicrobial properties) along with gluconic acid from glucose which helps in calcium absorption. Invertase converts sucrose to fructose and glucose. Dextrin and maltose are produced from long starch chains by the activity of amylase enzyme. Catalase helps in producing oxygen and water from hydrogen peroxide (32).

**Table 1:** Average composition of honey (31)

**Honey (Nutritional value per 100 g)**					
Component					Average
**Carbohydrates**					82.4 g
Fructose					38.5 g
Glucose					31 g
Sucrose					1 g
Other sugars					11.7 g
Dietary fiber					0.2 g
**Fat**					0 g
**Protein**					0.3 g
**Water**					17.1 g
Riboflavin (Vit. B_2_)					0.038 mg
Niacin (Vit. B_3_)					0.121 mg
Pantothenic acid (Vit. B_5_)					0.068 mg
Pyridoxine (Vit. B_6_)					0.024 mg
Folate (Vit. B_9_)					0.002 mg
Vitamin C					0.5 mg
Calcium					6 mg
Iron					0.42 mg
Magnesium					2 mg
Phosphorus					4 mg
Potassium					52 mg
Sodium					4 mg
Zinc					0.22 mg

*Physical properties of natural honey*

Honey has several important qualities in addition to composition and taste. Freshly extracted honey is a viscous liquid. Its viscosity depends on large variety of substances and therefore varies with its composition and particularly with its water content. Hygroscopicity is another property of honey and describes the ability of honey to absorb and hold moisture from environment. Normal honey with water content of 18.8% or less will absorb moisture from air of a relative humidity of above 60%. The surface tension of honey varies with the origin of the honey and is probably due to colloidal substances. Together with high viscosity, it is responsible for the foaming characteristics of honey (24).

The color in liquid honey varies from clear and colorless (like water) to dark amber or black. The various honey colors are basically all shades of yellow and amber. Color varies with botanical origin, age, and storage conditions, but transparency or clarity depends on the amount of suspended particles such as pollen (24). Less common honey colors are bright yellow (sunflower), reddish undertones (chest nut), grayish (eucalyptus) and greenish (honeydew). Once crystallized, honey turns lighter in color because the glucose crystals are white. Honey crystallization results from the formation of monohydrate glucose crystals, which vary in number, shape, dimension, and quality with the honey composition and storage conditions. The lower the water and the higher the glucose content of honey, the faster the crystallization (24). 

***Traditional uses of natural honey***

Human use of honey is traced to some 8000 years ago as depicted by Stone Age paintings (32). The ancient Egyptians, Assyrians, Chinese, Greeks and Romans employed honey for wounds and diseases of the gut (12). Here, some of the beneficial effects of honey which have been utilized by ancient races are summarized.

***Honey in*** ***i*** ***ndian system of*** ***a*** ***yurveda***

Ayurveda is a compound word i.e., *âyus* meaning ‘life’ or ‘life principle’, and the word *veda*, which refers to ‘a system of knowledge’. Hence ‘Ayurveda’ roughly translates as the ‘knowledge of life’ (33). The ancient *vedic* civilization considered honey one of nature^'^s most remarkable gifts to mankind. Traditionally, according to the texts of Ayurveda, honey is a boon to those with weak digestion. Also it has been emphasized that the use of honey is highly beneficial in the treatment of irritating cough. Honey is regarded by Ayurvedic experts, as valuable in keeping the teeth and gums healthy (34). It has been used for centuries for the treatment of insomnia because it has hypnotic action. Additionally, traditional Ayurvedic experts recommend honey for skin disorders (such as wounds and burns), cardiac pain and palpitation, all imbalances of the lungs and anemia. Honey has a long history of Ayurvedic use for various eye ailments. Applied daily to the eyes, it improves the eye-sight. Moreover, honey is regarded as useful in the prevention of cataract (34).

***Honey in ancient Egypt***

Honey was the most popular Egyptian drug being mentioned 500 times in 900 remedies (12). Its prescription for a standard wound salve discovered in the Smith papyrus (an Egyptian text dating from between 2600 and 2200 B.C.) calls for a mixture of mrht (grease), byt (honey) and ftt (lint/fibre) as transliterated from hieroglyphic symbols (35). Almost all Egyptian medicines contained honey together with wine and milk. The ancient Egyptians offered honey to their deities as a sacrifice (36). They also used honey for embalming the dead. Honey was utilized for its antibacterial properties that helped heal infected wounds. Moreover, honey was used as a topical ointment (31).

***Honey in ancient Greece***

Oenomel is an ancient Greek beverage consisting of honey and unfermented grape juice. It is sometimes used as a folk remedy for gout and certain nervous disorders (31). Hippocrates, the great Greek scientist, prescribed a simple diet, favouring honey given as oxymel (vinegar and honey) for pain, hydromel (water and honey) for thirst, and a mixture of honey, water and various medicinal substances for acute fevers (35). Also he utilized honey for baldness, contraception, wound healing, laxative action, cough and sore throat, eye diseases, topical antisepsis, prevention and treatment of scars (32).

***Honey in*** ***i*** ***slamic medicine***

In Islamic medical system, honey is considered a healthy drink. The holy Qur'an vividly illustrates the potential therapeutic value of honey: "And thy Lord taught the bee to build its cells in hills, on trees, and in (men’s) habitations; Then to eat of all the produce (of the earth), and find with skill the spacious paths of its Lord: there issues from within their bodies a drink of varying colors, wherein is healing for men: verily in this is a sign for those who give thought". Moreover, the Muslim prophet Mohammad (SA) recommended the use of honey for the treatment of diarrhea (37). Avicenna, the great Iranian scientist and physician, almost 1000 years ago, had recommended honey as one of best remedies in the treatment of tuberculosis (38).

*Place of honey in modern medicine*

***Antimicrobial properties of honey***

In addition to important role of natural honey in the traditional medicine, during the past few decades, it was subjected to laboratory and clinical investigations. Antibacterial activity of honey is one of the most important findings that was first recognized in 1892; by van Ketel (39). 

**Pathogens found sensitive to honey**

Honey has been reported to have an inhibitory effect to around 60 species of bacteria including aerobes and anaerobes, gram-positives and gram-negatives (24). Pathogens that are found to be sensitive to anti-infective properties of honey are manifold (40). Various results are in favor of its activity against *Bacillus anthracis,* *Corynebacterium diptheriae, Haemophilus* *influenzae, Klebsiella pneumoniae, Listeria* *monocytogenes, Mycobacterium tuberculosis, Pasteurella multicoda,* Yersinia enterocolitica*, Proteus species, Pseudomonas aeruginosa*, *Acinetobacter spp*, *Salmonella diarrhoea*, *Sal. typhi*, *Serratia marcescens, Shigella dysentery*, *Staphylococcus aureus, Streptococcus* *faecalis, Strep. mutans, Strep. pneumoniae, Strep. pyogenes and Vibrio* *cholerae* (15, 38, 32). Previously, a small number of case studies examining the antimicrobial activity of honey against *methicillin-resistant Staph. aureus (MRSA)* organisms demonstrated that natural honey had an antimicrobial activity against the *community-associated MRSA* organisms in *in vitro* condition (41-43). The MIC (minimum inhibitory concentration) of honey was found to range from 1.8% to 10.8% (v/v), i.e. the honey had sufficient antibacterial potency to still be able to stop bacterial growth if diluted at least nine times, and up to 56 times for *Staphylococcus aureus*, the most common wound pathogen (44). It has been indicated that diluted honey treated urinary tract infections because certain bacteria causing urinary tract infections, e.g. *E. coli*, *Proteus* species and *Strep. faecalis*, were found to be sensitive to the antibacterial activity of honey (45).

*In vitro *studies of *H. pylori* isolates which cause gastritis have been shown to be inhibited by a 20% solution of honey. Even isolates that exhibited a resistance to other antimicrobial agents were susceptible (10, 15). Unlike most conventional antibiotics, it has been reported that honey dose not lead to development of antibiotic-resistant bacteria, and it may be used continuously (14).

Honey can act as both bacteriostatic and bactericidal depending on the concentration used. Pasture honey (4-8% ) and 5-11% manuka honey were bacteriostatic whereas bactericidal activity was achieved at 5-10% and 8- 15% (v/v) concentrations, respectively. In contrast, artificial honey (sugar solution which mimics composition of honey) was bacteriostatic only (at 20- 30%) and not bactericidal (32).

**Possible mechanisms of antimicrobial activity of honey**

Mechanisms of antimicrobial activity of honey are different from antibiotics, which destroy the bacteria’s cell wall or inhibit intracellular metabolic pathways. The antibacterial activity is related to four properties of honey. First, honey draws moisture out of the environment and thus dehydrates bacteria. The sugar content of honey is also high enough to hinder the growth of microbes, but the sugar content alone is not the sole reason for honey’s antibacterial properties (46). Second, the pH of honey is between 3.2 and 4.5, and this acidity is low enough to inhibit the growth of most microorganisms. Hydrogen peroxide produced by the glucose oxidase is the third and probably the most important antibacterial component, although some authors believe the nonperoxide activity to be more important. Lastly, several phytochemical factors for antibacterial activity have been identified in honey (13, 14).

Hydrogen peroxide, glucose oxidase, catalase, phytochemical factors have been described as non-peroxide antibacterial factors (24). In addition volatiles, organic acids, lysozyme, beeswax, nectar, pollen and propolis are important chemical factors that provide antibacterial properties to honey (32, 47, 48). Honey also contains oligosaccharides in small quantities. Shin & Ustunol (2005) related the sugar composition of honey from different floral sources to the growth inhibition of various intestinal bacteria (49, 50). Moreover, it is reported that a part of the antibacterial activity might be attributed to the components of plant origin (47). All these physical and chemical factors give honey unique properties as a wound dressing: it has a rapid clearance of infections, rapid debridement of wounds, rapid suppression of inflammation, minimization of scarring, and stimulation of angiogenesis as well as tissue granulation and epithelium growth (50, 51).

***Wound healing***

One of the most studied and most effective uses of honey is found in healing of wounds (17). The Russians used honey in World War I to prevent wound infection and to accelerate wound healing. The Germans combined cod liver oil and honey to treat ulcers, burns, fistulas and boils (32). Nearly all types of wounds like abrasion, abscess, amputation, bed sores /decubitus ulcers, burns, chill blains, burst abdominal wound, cracked nipples, fistulas, diabetic, malignant, leprosy, traumatic, cervical, varicose and sickle cell ulcers, septic wounds, surgical wound or wounds of abdominal wall and perineum are found to be responsive to honey therapy. Application of honey as wound dressing leads to stimulation of healing process and rapidly clears the infection. Honey has cleansing action on wounds, stimulates tissue regeneration and reduces inflammation. Honey impregnated pads act as non adhesive tissue dressing (32, 52, 53).

The exact molecular mechanism of wound healing using honey is yet to be elucidated. However, several recommendations are made regarding appropriate wound dressing with honey. Type of wound and degree of severity will affect efficacy. Selected honey should be used in sufficient quantities so that it remains there if diluted with wound exudates. It should cover and extend beyond the wound margins. Better results occur when applied on dressing than on wound. All the cavities should be adequately filled with honey and occlusive dressing applied to prevent oozing from the wound (32, 54, 55). On burns, it has an initial soothing and later rapid healing effects. It has been used as wound barrier against tumor implantation in laparoscopic oncological surgery. No infection has been reported from the application of honey to open wounds. It has a potential therapeutic role in the treatment of gingivitis and periodontal disease (56). In one of cases of knee amputation in a young boy, which was heavily infected with *Pseudo.* and *Staph. aureus *and non responsive to conventional treatment, application of sterilized active manuka honey dressing pads led to complete healing in ten weeks (57). Similar results are found with burns. Honey dressing speeds up healing process, sterilizes wound and reduces pain (58). Studies in Fournier’s gangrene showed rapid improvement with decreased edema and discharge, rapid regeneration and little or no scarring, effective wound debridement and a decrease in mortality (59).

Honey is used successfully for treating ulcerations following radical surgery for carcinoma of the breast and varicose veins. It is also used following radical surgery for carcinoma of vulva resulting in infection free wound with minimal wound debridement and hospital stay (60). In patients with postoperative wound infections following caesarean section or hysterectomies, topical honey application causes faster eradication of bacterial infections, reduces antibiotic use and hospital stay, accelerates wound healing, and results in minimal scar formation (53). Similar efficacy is observed in bed sores and decubitus ulcers (61).

Clinical trials are conducted comparing honey dressing in burns with amniotic membrane dressing; silver sulfadiazine dressing and boiled potato peel dressing. Honey dressing showed better improvement in these cases and showed early healing with lesser degree of contracture and scarring (32, 45). Good histological preservation of skin grafts after honey treatment has also been described (62). Motallebnejad et. al. (2008) reported that application of natural honey is effective in managing radiation induced mucositis (63). 

An unusual application of honey was its use as a means of confirming the presence of measles during its early stages. Honey is reportedly massaged onto the eruptions which then, in the case of measles, become more pronounced on the following day. Continued application of honey is performed until total disappearance of the eruptions occurs (45). 

**Advantages of honey as wound dressing**

The remarkably rapid effect of honey in cleaning up wounds is due to a combination of the osmotic outflow and a bioactive effect of honey. The enzyme glucose oxidase of honey provides glucose to leucocytes, which is essential for respiratory burst to produce hydrogen peroxide leading to antibacterial activity of macrophages. The acidity of honey further aids in antibacterial activity (52). Presences of a wide range of amino acids, vitamins and trace elements also have direct nutrient effect on regenerating tissues. Osmotic outflow after the application of honey assists in lifting dirt and debris from the bed of the wound. The dressing thus is non-sticky and enables pain free change. Some people have however experienced pain or discomfort. This may be because of naked nerve endings coming in contact with acidity of honey (32). The clearing of infection seen when honey is applied to a wound may reflect more than just antibacterial properties. Recent research shows that the proliferation of peripheral blood B-lymphocytes and T-lymphocytes in cell culture is stimulated by honey at concentrations as low as 0.1%; and phagocytes are activated by honey at concentrations as low as 0.1% (24). In one study, natural honey significantly increased the tumor necrosis factor-α (TNF-α), interleukin (IL)-1β and IL-6 release from MonoMac-6 cells (and human monocytes) which activate the immune response to infection. Therefore, it was suggested that the effect of honey on wound healing may in part be related to the stimulation of inflammatory cytokines from monocytic cells (18, 25, 64, 65). In addition, honey dressing has economic advantages to the patient. Rapid healing reduces hospital stay and dressing material and surgical costs (35).

***Gastrointestinal tract diseases***

Oral administration of honey to treat and protect against gastrointestinal infection such as gastritis, duodenitis and gastric ulceration caused by bacteria and rotavirus has been reported (66-70). Attachment of bacteria to mucosal epithelial cells is considered the initial event in the development of bacterial infections of the gastrointestinal tract. Blocking attachment of pathogenic microorganisms to the intestinal epithelium represents a potential strategy for disease prevention. Alnaqdy *et al* (2005) demonstrated that the prevention of bacterial adherence caused by honey was through effect on bacteria, rather than epithelial cells. There are several possible explanations for prevention of bacterial adherence demonstrated by honey: (a) non-specific mechanical inhibition perhaps through the coating of the bacteria by the honey; (b) some of the fractions, within honey, may alter bacterial electrostatic charge or hydrophobicity which have been reported to be important factors in the interaction of bacteria with host cells (70-72) or (c) killing of the bacteria due to the previously mentioned antibacterial factors in honey (70).

Diarrhea and gastroenteritis are found to resolve quickly with honey (32, 67, 73). At 5% (v/v) concentration, honey decreased the duration of diarrhea in cases of bacterial gastroenteritis as compared to group using sugar in replacement fluid. No change was seen in viral gastroenteritis. In rehydration fluid, honey adds potassium and water uptake without increasing sodium uptake. It also helps to repair the damaged intestinal mucosa, stimulates the growth of new tissues and work as an anti-inflammatory agent (32). Nasutia *et al* (2006) demonstrated that oral pretreatment of honey (2 g/kg), prevented indomethacin-induced gastric lesions, microvascular permeability, and myeloperoxidase activity of the stomach (74). *H. Pylori* is found to be sensitive to honey with a median level of antibacterial activity due to the presence of hydrogen peroxide at a 20% concentration (32, 68). 

For evaluation of gastric cytoprotective properties of natural honey, perfusion of the stomach with isotonic honey resulted in a marked reduction of the area of the lesions caused by ethanol (75). Also, it has been suggested that natural honey has curative properties for healing of antral ulcers and may be used like sucralfate in the management of peptic ulcer disease (76). 

***Fungal infections***

Honey has been reported to have inhibitory effects on fungi. Pure honey inhibits fungal growth and diluted honey appears capable of inhibiting toxin production (13). An antifungal action has also been observed for some yeast and species of Aspergillus and Penicillium, as well as all the common *dermatophytes* (25, 77). Candidiasis, caused by *Candida albicans*, may respond to honey (78, 32). Cutaneous and superficial mycoses like ringworm and athletes foot are found to be responsive to honey. This responsiveness is partly due to the inhibition of fungal growth and partly to inhibition of bacterial infection (32). In addition, some studies have reported that topical application of honey was effective in treating seborrheic dermatitis and dandruff (53, 79). 

***Antiviral effects of honey***

In addition to antibacterial and antifungal effects, natural honey has showed antiviral effect. Al-Waili (2004) investigated the effect of the topical application of honey on recurrent attacks of herpes lesions and concluded that topical honey application was safe and effective in the management of the signs and symptoms of recurrent lesions from labial and genital herpes compared to acyclovir cream (80)**.** Honey has also been reported to have inhibitory effects on rubella virus activity (13).

***Ophthalmology and honey***

Honey is used worldwide for the treatment of various ophthalmological conditions like blepharitis, keratitis, conjunctivitis, corneal injuries, chemical and thermal burns to eyes (45, 81). In one study, with topical application of honey as ointment, in 102 patients with non responsive eye disorders, improvement was seen in 85% patients and in remaining 15% there was no disease progression. Application of honey in infective conjunctivitis reduced redness, swelling, pus discharge and time to bacterial eradication (32, 78, 80).

***Honey*** ***as a carbohydrate source***

Honey is a natural mixture of fructose-glucose along with some oligosaccharides, proteins, vitamins and minerals. Some studies demonstrated that honey is an effective carbohydrate source for athletes before and after resistance training and during endurance exercise (32).

***Honey and diabetes***

The use of honey in type I and type II diabetes was associated with significantly lower glycemic index than with glucose or sucrose in normal diabetes. Honey compared with dextrose caused a significantly lower rise in plasma glucose levels in diabetic subjects. It also caused reduction of blood lipids, homocysteine levels and C-reactive protein (CRP) levels in normal and hyperlipidemic subjects (32, 40). In earlier observations, it was found that honey stimulates insulin secretion, decrease blood glucose levels, elevates hemoglobin concentration and improves lipid profile (13). 

***Honey as food preservative and prebiotic***

Hydrogen peroxide and non peroxide components such as antioxidants are found to inhibit growth of *Shigella, Listeria monocytogenes*, and *Staph. aureus *helping in food preservation. *Clostridium botulinum *however may be present in small amounts in honey. It has a good potential to be used as a natural source of antioxidants to reduce negative effects of polyphenol oxidase browning in fruit and vegetable processing (32, 82).

A prebiotic is a non-digestible dietary supplement that modifies the balance of the intestinal microflora stimulating the growth and activity of the beneficial organisms and suppressing potentially deleterious bacteria. Honey is found to be a suitable sweetener in fermented milk products without inhibiting the growth of common bacteria like *Strep.* *thermophilus, Lactobacillus acidophilus,* *Lacto. delbruekii and Bifidobacterium bifidum *which are important for maintaining the health of gastrointestinal tract. Honey also increased and supported the growth of *bifidobacterium* (32, 83), which is mainly due to the presence of a variety of oligosaccharides (1, 11, 32).

***Anti-inflammatory effects of honey***

In a recent investigation, it was reported that honey reduces the activities of cyclooxygenase-1 and cyclooxygenase-2, thus showing anti-inflammatory effects (84). Honey also demonstrates immunomodulatory activities (85). Moreover, ingestion of diluted natural honey showed reduction effect on concentrations of prostaglandins such as PGE_2_, PGF_2α_ and thromboxane B_2_ in plasma of normal individuals (19)). Lesions treated with honey show less edema, infiltration of fewer granular and mononuclear cells, less necrosis, better wound contraction, improved epithelization and low glycosaminoglycan and proteoglycan concentrations. Moreover, it reduces inflammation and exudation, promotes healing, diminishes scar size and stimulates tissue regeneration (19). Honey has also been reported to treat eczema, psoriasis and dandruff (19, 85). In an inflammatory model of colitis, honey was as effective as prednisolone treatment (16 ). Drugs for treating inflammation have serious limitations: corticosteroids suppress tissue growth and suppress the immune response, and the non-steroidal anti-inflammatory drugs are harmful to cells, especially in the stomach. But honey has an anti-inflammatory action free from adverse side effects (44 ). Our recently generated and unpublished laboratory data suggests that honey is able to inhibit inflammatory parameters, angiogenesis as well as showing potent inhibitory activities against TNF-α a and PGE_2_ in air pouch model of inflammation.

***Antioxidant activity of honey***

Today, we know well that radicals cause molecular transformations and gene mutations in many types of organisms. Oxidative stress is well-known to cause many diseases (86 ), and scientists in many different disciplines became more interested in natural sources which could provide active components to prevent or reduce its impacts on cells (47 , 87 , 88).

Natural honey contains many flavonoides (such as apigenin, pinocembrin, kaempferol, quercetin, galangin, chrysin and hesperetin), phenolic acids (such as ellagic, caffeic, p-coumaric and ferulic acids), ascorbic acid, tocopherols, catalase, superoxide dismutase, reduced glutathione, Maillard reaction products and peptides. Most of the above compounds work together to provide a synergistic antioxidant effect (4 - 6). Hence, it has been suggested that honey, as a natural antioxidant, may serve as an alternative to some preservatives such as sodium tripolyphosphate in food preservation to delay lipid oxidation (4).

The botanical origin of honey has the greatest influence on its antioxidant activity, while processing, handling and storage affect honey antioxidant activity only to a minor degree (7, 89-92). The antioxidant activity is strongly correlated with the content of total phenolics (7, 45, 9 , 92– 94). Beside this, a strong correlation was found between antioxidant activity and the color of honey. Many researchers found that dark honey has a higher total phenolic content and consequently a higher antioxidant capacity (89 , 92 , 95 ). Blasa *et al *(2007) showed that the antioxidant activity was located in both the ether and the water fractions, indicating that the flavonoids of honey may be available to various compartments of the human body where they may exert different physiological effects (96 ).

**Properties of phenolic compounds of honey**

Phenolic compounds are one of the most important groups of compounds occurring in plants, comprising at least 8000 different known structures (48, 97). These compounds are reported to exhibit anticarcinogenic, anti-inflammatory, antiatherogenic, antithrombotic, immune modulating and analgesic activities, among others and exert these functions as antioxidants (48, 98). The phenolic compounds of honey are phenolic acids and flavonoids, which are considered potential markers of the botanical origin of honey (7, 48, 99). The antioxidant activities of phenolics are related to a number of different mechanisms, such as free radical-scavenging, hydrogen-donation, singlet oxygen quenching, metal ion chelation, and acting as a substrate for radicals such as superoxide and hydroxyl (47).

***Cardiovascular diseases***

Ischemic heart disease (IHD) causes more deaths and disability and incurs greater economic costs than any other illness in the developed world (100). Arrhythmias and myocardial infarction (MI) are serious manifestations of IHD. In the course of cardiac surgery and MI, ventricular arrhythmias such as ventricular tachycardia and ventricular fibrillation are the most important causes of mortality (100). In management of such conditions, drug therapy (especially anti-arrhythmic drugs) may be lifesaving. On the other hand, the hazards of anti-arrhythmic drugs (such as lethal arrhythmias in some patients) have led to a limitation on the administration of anti-arrhythmic drugs (101). Hence, there is a tendency to use drugs which have less adverse effects and more efficacies. Natural honey has been applied for medicinal purposes since ancient times (102), however, in the case of cardiovascular diseases, most of the previous studies were carried out in animals and mainly focused on honey's effects against cardiovascular risk factors such as hyperlipidemia and production of free radicals (103-106 ). Antioxidants present in honey include Vitamin C, monophenolics, flavonoids, and polyphenolics. Regular flavonoid intake is associated with a reduced risk of cardiovascular diseases. A wide range of phenolic compounds is present in honey which has promising effect in the treatment of cardiovascular diseases. In coronary heart disease (CHD), the protective effects of phenolic compounds include mainly antithrombotic, anti-ischemic, anti-oxidant, and vasorelaxant. It is suggested that flavonoids decrease the risk of CHD by three major actions: improving coronary vasodilatation, decreasing the ability of platelets in the blood to clot, and preventing LDLs from oxidizing (107). In 38 overweight individuals, the effect of natural honey on total cholesterol, LDL-C, high-density lipoprotein cholesterol (HDL-C), triacylglycerole, C-reactive protein (CRP), fasting blood glucose and body weight were investigated. The results showed that receiving 70 g of natural honey for 30 days caused reduction in total cholesterol, LDL-C, triacylglycerole and CRP (*P*<0.05). The authors concluded that natural honey reduces cardiovascular risk factors, particularly in subjects with elevated risk factors, and it does not increase body weight in overweight or obese subjects (106). The effects of ingestion of 75 g of natural honey compared to the same amount of artificial honey (fructose plus glucose) were studied in humans. Elevation of insulin and CRP was significantly higher after glucose intake than after honey consumption. In addition, honey reduced cholesterol, LDL-C, and TG and slightly elevated HDL-C. In patients with hypertriglyceridemia, artificial honey increased TG, while honey decreased TG. In patients with hyperlipidemia, artificial honey increased LDL-C, while honey decreased LDL-C. In diabetic patients, honey compared with dextrose caused a significantly lower rise of plasma glucose. Honey can contain nitric oxide (NO) metabolites and increased levels of NO in honey might have a protecting function in cardiovascular diseases (108). 

Honey also decreased venous blood pressure, which can reduce the preload of the heart and consequently may diminish the congestion in the venous system (6).

Najafi *et al *(2008) have demonstrated prophylactic effects of natural honey as a pharmacologic preconditioning agent on ischemia/reperfusion (I/R) induced injuries, where short-term perfusion of enriched Krebs solution with natural honey for 10 min before to 10 min after ischemia were perfused in isolated rat heart (109). The results of another *in vitro* study showed that chronic oral administration of natural honey (for 45 days) produces potent anti-arrhythmic and anti-infarction effects in rat (110). In a study, pretreatment of anesthetized normal or stressed rats with natural honey (5 g/kg) for 1 hr prior to adrenaline injection (100 mcg/kg) could protect them from epinephrine-induced vasomotor dysfunction and cardiac disorders and preserved the positive inotropic effect of adrenaline. The authors concluded that natural honey might cause its cardioprotective and therapeutic effects against adrenaline-induced cardiac and vasomotor dysfunction directly (*via *its high total antioxidant capacity and enzymatic and non-enzymatic antioxidants, besides its substantial quantities of mineral elements such as magnesium, sodium, and chlorine), and/or indirectly by stimulating release of nitric oxide from endothelium through the influence of vitamin C (6). 

Honey also inhibited oxidative stress which may be partly responsible for its neuroprotective activity against *in vitro* cell death and *in vivo* focal cerebral ischemia (111). Regarding the existence of many organic compounds with antioxidant and radical-scavenging activity in honey composition, it seems that honey has the potential capability to serve as an important source of natural antioxidants in human nutrition (112). In addition, regarding anti-inflammatory effect, honey causes a reduction in necrosis tissues (38, 113). 

***Other effects of honey***

Positive effect of honey as an anticarcinogenic agent is reported in some studies (32, 114, 115). Honey has showed antineoplastic activity in the experimental bladder cancer (22). Natural honey can play an important role in the treatment of chest pain, fatigue and vertigo. This is probably due to the high nutritional energy content of honey which provides immediately available calories after consumption (45). Benefits of honey also have been seen in tooth extraction pain and infection or caries due to radiation-induced xerostomia (32,114, 115). It has been shown that honey is a very effective agent for split thickness skin graft fixations and can be easily used (14). In a survey that was performed in central Burkina Faso, it was found that local inhabitants use honey therapeutically for treatment of respiratory ailments, measles, period pains, postnatal disorders, male impotence and pharyngitis due to its antibacterial and anti-inflammatory effects (45). Honey has also been reported to exhibit anti-leishmanial effects *in vitro *(13).

In one study, daily consumption of honey showed a variety of beneficial effects on hematological indices, blood levels of minerals and enzymes and endocrine system (85). In another study, oral honey stimulated antibody production during primary and secondary immune responses against thymus-dependent and thymus-independent antigens (13). 

According to Guerrini *et al *(2009) stingless bee honey acts as a protective agent against DNA damage and could represent interesting evidence in relation to the determined antioxidant capacity (116). Kilicoglu *et al* (2008) examined the effects of honey on oxidative stress and apoptosis in experimental obstructive jaundice and found that honey diminished the negative effects of bile duct ligation on the hepatic ultrastructure. This effect might be due to its antioxidant and anti-inflammatory activities (117). In another study, administration of honey investigated on *N*-ethylmaleimide (NEM-induced) liver injury in rats. NEM is a sulphydryl blocker which impairs the sulphydryl dependent antioxidant system (mainly glutathione) in the body. The findings imply that depletion of glutathione concentration plays a causal role in NEM-induced liver injury, and that the hepatoprotective effect of honey may be mediated through sulfhydryl-sensitive processes (118). In hepatocellular carcinoma honey can be considered as promising with inhibition of the proliferation, protease activity and gelatinase activity of HepG2 cells in an independent manner (119).

Zaid *et al* (2010) showed that honey had beneficial effects on menopausal rats by preventing uterine atrophy, increased bone density and suppression of increased body weight. They concluded that honey could be an alternative to hormone replacement therapy (120). 

***Adverse effects of honey***

Honey is relatively free of adverse effects. Topical application of honey may lead to transient stinging sensation. Otherwise it is described in different forms as soothing, relieving pain, to be non-irritating and a painless dressing change. Allergy to honey is rare, but there could be an allergic reaction to either pollen or bee proteins in honey. Excessive application of honey may lead to dehydration of tissues which can however be restored by saline packs. Theoretical risk of rise in blood glucose levels may always be there when applied to large open wound in diabetics. Risk of wound botulism, due to presence of spores of *Clostridia*, can be minimized by gamma irradiation which will kill the spores of clostridia without any loss of antibacterial activity (32, 121).

***Concluding remarks***

To date, researchers pay more attention to medicines with natural origin and believe that natural products may be efficient therapeutics in comparison with the synthetic drugs. One of the most important natural products is honey, which has been used for different medicinal purposes since ancient times. In addition to important role of honey in the traditional medicine, scientists also accept honey as a new effective medicine for many kinds of diseases. The most well known effect of honey is antibacterial activity. Honey has also been reported to exhibit an inhibitory effect on yeast, fungi, leishmania and some viruses. Topical application of honey has been effectively used on mucocutaneous injuries such as genital lesions, superficial skin burns and post operation wounds. In addition, honey has been used in some gastrointestinal, cardiovascular, inflammatory and neoplastic states. The antioxidant capacity of honey which plays an important role in its useful effects, related to a wide range of compounds including phenolics, peptides, organic acids, enzymes, and Maillard reaction products. 
